# Operative versus non-operative management for perforated peptic ulcer disease

**DOI:** 10.1016/j.amsu.2022.104643

**Published:** 2022-09-13

**Authors:** Sapna Gupta, Awad Ali Alawad, Kimberly Dacosta, Adel Mahmoud, Taghreed Mohammed

**Affiliations:** a Safdarjung Hospital, New Delhi, India; b University of Medical Sciences and Technology, Khartoum, Sudan; c University Hospital of Wales, Cardiff, UK; d University Hospital Southampton NHS Trust, Southampton, UK; e Tabuk Hospital, Tabuk, Saudi Arabia

**Keywords:** Perforated peptic ulcer, Management, Operative versus non-operative

## Abstract

Perforated peptic ulcer (PPU) treatment guidelines are still up for discussion. Due to the morbidity and mortality linked to each, the use of both operative and non-operative management, including conservative and endoscopic treatment, is still debatable. A standardized protocol has been used to write a best evidence topic. The discussion focused on whether operative management for PPU is preferable to non-operational management or vice versa.

MEDLINE, the Cochrane Library, Scopus, and the Web of Science were the databases used to conduct an electronic search of the pertinent literature.

We found 56 articles, out of these only 5 studies were found to be appropriate to answer the question. The outcome assessed was failure of management. The best evidence showed that both operative and non-operative management can be used with similar outcomes depending on the patient selection for each category.

## Highlights

• Operative versus non-operative management of perforated peptic ulcer disease.
•Non-operative management is reserved for selective patients for perforated peptic ulcer.
•Endoscopic treatment and interventional radiology can serve as a bridge between conservative and surgical management

## 1 Introduction

A best evidence topic (BET) provides evidence-based solutions to frequent clinical issues through a structured technique of literature review. The International Journal of Surgery's design was used to build this BET [1]. This framework was used because a previous literature assessment indicated that the quality of the information available was insufficient to carry out a useful systematic review or meta-analysis.

## 2 Clinical scenario

A 65-year-old male patient with history of arthritis and ischemic heart disease presented to accident and emergency department with sudden onset upper abdominal pain. Investigations confirmed localised perforated duodenal ulcer. Surgical team looking after the patient is debating whether to manage this operatively or non-operatively based on best evidence available.

## 3 Three-part question

Does [operative management ] preferred over [non-operative management] in patients with [perforated peptic ulcer]?

## 4 Search strategy

A. Embase 2002 to October 2021 using the OVID interface:
[perforated peptic ulcer] AND [management] AND [operative] AND [non-operative] OR [conservative]
B. Medline using the PubMed interface:
[perforated peptic ulcer] AND [management] AND [operative] AND [non-operative].

The results were limited to English articles and human studies.

## 5 Search outcome

There were 56 articles that might be relevant. After eliminating duplicate references and unrelated literature, twelve candidate articles remained. After carefully reading the complete contents of these articles, five papers were selected to give the most convincing supporting information to address the subject.

## 6 Result

See the table.

**Table:** 

Author, date of publication, journal and country	Study type	Patient group	Outcomes Follow up	Key results	Additional comments
Koray Karabulut et al. 2015 Turkish Journal of Trauma and Emergency Surgery Turkey	Retrospective Cohort Study Level III	Total of 41 patients Group 1- Operative treatment (35) Group 2- Non-operative treatment (6)	Primary end point- Failure of management (FM)	FM rate was Group 1- 0% Group 2- 0%	Single Centre Retrospective study Small sample size
Negm et al. 2022 World Journal of Emergency Surgery Egypt	Prospective Randomised control trial Level I b	Total of 100 patients Group 1- Operative treatment (50) Group 2-Non-operative treatment (50)	Primary end point- Failure of management- Leak rate (LR)	Leak rate (LR) Group 1–5% Group 2 - 2%	Randomised prospective study Single Centre Small sample size
Feng Ciao et al. 2013 Asian Journal of Surgery China	Retrospective Cohort Study Level III	Total of 241 patients Group 1- Operative treatment (134) Group 2- Non-operative treatment (107)	Primary end point- Failure of management (FM)- Abdominal abscess post management	Abdominal abscess post management Group 1–1.5% Group 2–3.7%	Single Centre Retrospective study Large sample size
Jorge Vázquez et al. 2021 Surgical Endoscopy Sweden	Retrospective Randomised control trial Level I b	Total of 28 patients Group 1- Operative treatment (15) Group 2- Non-operative treatment (13)	Primary end point- Failure of management (FM)- Post management complications	Post management complications Group 1–40.0% Group 2–53.8%	Multicentre retrospective randomised study Small sample size
Md Rahuman et al. 2003 Ceylon Medical Journal Bangladesh	Retrospective Cohort Study Level III	Total of 491 patients Group 1- Operative treatment (364) Group 2- Non-operative treatment for healthy patients presenting early (56) Group 3- Group 2- Non-operative treatment For elderly patients presenting late (71)	Primary end point- Failure of management (FM)- Post management complications	Post management complication rate Group 1–35.9% Group 2 and 3 combined- 14.1%	Retrospective study Single centre Large sample size

## 7 Discussion

Perforated peptic ulcer (PPU) is a common surgical emergency. Over the last 50 years, although there has been a dramatic fall in the prevalence of peptic ulcer disease (PUD) due to use of treatment for elimination of Helicobater pylori, the number of patients needing surgery has remained mostly unchanged [2,3].

The viability of nonoperative care for PPU was not demonstrated until the results of the first randomised controlled trial were published in 1989. According to Crofts et al. [4], nonoperative therapy using comparable methods can effectively treat approximately 72% of cases with comparable morbidity and mortality as compared to the immediate surgical group. Perforated peptic ulcer is currently treated medically and surgically, both of which have benefits and drawbacks of their own. Determining the optimal course of treatment for the patient can be challenging for the clinician as a result.

Medical management for PPU include intravenous antibiotics, nil by mouth and a nasogastric tube, anti-secretory and anti-acid medication (PPIs) and a water-soluble contrast imaging study to confirm a sealed leak [5]. Many studies in the past have demonstrated that PPU can seal spontaneously with successful non-operative strategy [6,7]. Cao et al. [7] have demonstrated that degree of secondary peritonitis should be the primary consideration when choosing the treatment strategy because it poses a greater risk to the patients' lives than the peptic ulcer itself. In patients with few or isolated symptoms and is in good clinical health non-operative management must be considered. However, consideration should be given to the reported mortality increase that occurs with every hour of delay to surgery [[8], [9], [10], [11]].

Endoscopic treatment e.g. clips or stents presents an alternative between non-operative and operative treatment. Although not used routinely, some studies have demonstrated lower mortality rate in patients undergoing endoscopic management and were discharged without additional surgical procedures [12,13].

Options for surgical treatment for PPU include open surgery or laparoscopic surgery. Laparoscopy is not yet supported by any evidence that it is superior to open surgery or that it is hazardous to individuals who have sepsis or widespread peritonitis [5], hence both surgically approaches are widely used based on surgeon's experience.

Studies included in our review are to help identifying best evidence practice for patients with PPU. Karabulut et al. [14] in their retrospective study with 41 patients demonstrated that the length of stay was similar in patients treated with conservative or surgical management. Conservative approach was suggested for patient with normal vital parameters and no signs of generalised peritonitis, while surgical treatment was for patients demonstrating sins of sepsis and peritonitis.

A prospective randomised control trial of 100 patients by Negm et al. [15] comparing endoscopic treatment with surgical treatment concluded that in high-risk surgical patients, combined endoscopic and interventional radiological drainage can effectively treat acute perforated peptic ulcer without the requirement for general anaesthesia, with minimal to no additional morbidity or mortality.

Another study by Cao et al. [7] demonstrated that nonoperative management was effective and safe in selected patients without peritonitis with similar length of hospital stay as patients with operative management.

A prospective randomised study of 43 patients by Vazquez et al. [16] demonstrated that, a safe alternative to conventional surgical closure for perforated duodenal ulcers is stent treatment in conjunction with laparoscopic lavage and drainage. Stent treatment is also a good alternative in cases of suture-line leakage.

Study by Rahuman-et al [17] with 491 patients case selection is important for various treatment options. Low risk, healthy patients can be treated with conservative management. For high risk patients elderly patients with multiple co-morbidities percutaneous abdominal drainage is suitable whereas medium risk patients with peritonitis should be treated with surgery.

In conclusion, the evidence of an optimal strategy for patients with PPU is currently based on a few studies most of which are retrospective single-institution studies. The use of non-operative therapy in patients who are healthy with minimal disease as well as in frail patients is a topic of current controversy. Large-scale research may yield greater information on the patient selection for conservative treatment, which is currently understudied.

## 8 Clinical bottom line

According to one prospective randomised controlled trial, one retrospective randomised control trail and 3 retrospective cohort studies there is no statistically significant difference between operative and non-operative management for perforated peptic ulcer.

### Limitation of this review

1. Small sample size in some studies
2. Four out of fiver studies were single centre
3. Most studies were retrospective rather than prospective

## Ethical approval

Not applicable.

## Sources of funding

No sources of funding

## Author contribution

Sapna Gupta: conducted the literature search and wrote the paper. Awad Alawad: assisted in the literature search and writing of paper. Kimberly DaCosta: assisted in writing of paper. Adel Mahmoud: assisted in writing of paper. Taghreed Mohammed: assisted in writing of paper.

## Trial registry number

1.Name of the registry:

2.Unique Identifying number or registration ID:

3.Hyperlink to your specific registration (must be publicly accessible and will be checked):

## Guarantor

Sapna Gupta.

## Consent

Not applicable.

## Provenance and peer review

Not commissioned, externally peer reviewed.

## Declaration of competing interest

No conflicts of interest.
